# Differential profiling of breast cancer plasma proteome by isotope-coded affinity tagging method reveals biotinidase as a breast cancer biomarker

**DOI:** 10.1186/1471-2407-10-114

**Published:** 2010-03-26

**Authors:** Un-Beom Kang, Younghee Ahn, Jong Won Lee, Yong-Hak Kim, Joon Kim, Myeong-Hee Yu, Dong-Young Noh, Cheolju Lee

**Affiliations:** 1 Life Sciences Division, Korea Institute of Science and Technology, Seoul 136-791, Korea; 2 Functional Proteomics Center, Korea Institute of Science and Technology, Seoul 136-791, Korea; 3 School of Life Sciences and Biotechnology, Korea University, Seoul 136-701, Korea; 4 Cancer Research Institute, Seoul National University College of Medicine, Seoul 110-744, Korea

## Abstract

**Background:**

Breast cancer is one of the leading causes of women's death worldwide. It is important to discover a reliable biomarker for the detection of breast cancer. Plasma is the most ideal source for cancer biomarker discovery since many cells cross-communicate through the secretion of soluble proteins into blood.

**Methods:**

Plasma proteomes obtained from 6 breast cancer patients and 6 normal healthy women were analyzed by using the isotope-coded affinity tag (ICAT) labeling approach and tandem mass spectrometry. All the plasma samples used were depleted of highly abundant 6 plasma proteins by immune-affinity column chromatography before ICAT labeling. Several proteins showing differential abundance level were selected based on literature searches and their specificity to the commercially available antibodies, and then verified by immunoblot assays.

**Results:**

A total of 155 proteins were identified and quantified by ICAT method. Among them, 33 proteins showed abundance changes by more than 1.5-fold between the plasmas of breast cancer patients and healthy women. We chose 5 proteins for the follow-up confirmation in the individual plasma samples using immunoblot assay. Four proteins, α1-acid glycoprotein 2, monocyte differentiation antigen CD14, biotinidase (BTD), and glutathione peroxidase 3, showed similar abundance ratio to ICAT result. Using a blind set of plasmas obtained from 21 breast cancer patients and 21 normal healthy controls, we confirmed that BTD was significantly down-regulated in breast cancer plasma (Wilcoxon rank-sum test, *p *= 0.002). BTD levels were lowered in all cancer grades (I-IV) except cancer grade zero. The area under the receiver operating characteristic curve of BTD was 0.78. Estrogen receptor status (*p *= 0.940) and progesterone receptor status (*p *= 0.440) were not associated with the plasma BTD levels.

**Conclusions:**

Our study suggests that BTD is a potential serological biomarker for the detection of breast cancer.

## Background

Breast cancer is one of the most common cancers in women worldwide [1]. Early detection and treatment of breast cancer in patients showed good prognosis, but current diagnostic techniques such as mammography, MRI and PET are not sufficient to detect early stages of breast cancer efficiently [2]. Finding a series of relevant biological markers for early cancer detection and diagnosis and monitoring the therapeutic response can definitely improve our ability to manage breast cancer [3-5].

An emerging issue of proteomics is to discover novel biological markers that can be applied to early detection, disease diagnosis and prediction of response to therapy [6]. Proteomics has advanced direct profiling of differentially expressed proteins between diseased and control samples, or at various stages of diseases under particular environments [7], and thus become a key technology in biomarker development pipeline. The biomarker pipeline can be divided into four phases: discovery, qualification, verification and validation phases [8]. Discovery phase is an unbiased and semiquantitative process, usually comprising simple binary comparisons between diseased and normal state. Tissues, body fluids, or even model cell lines are being utilized as proteome sources. The 'products' of the discovery phase are confirmed in the next qualification phase. Immunoassays with commercially available antibodies can be used. In verification phase, the analysis is extended to a larger number of samples, now incorporating a broader range of cases and controls. Although any proteome source can be used in the discovery phase, biomarkers that are detected and validated in specimens obtained by less invasive techniques, such as plasma or serum, are more desirable [8,9]. The blood serum or plasma contains enormous complexity of biological components which reflect spatio-temperal changes of diseased cells, tissues, or organs [10]. Knowing any change in the containment of blood caused by a specific disease like cancer will help us understand and develop detection and further management of the disease.

The objective of our study is to discover new breast cancer biomarkers using blood plasma as proteome sources. We previously analyzed breast cancer tissues [11] and secretome from a breast cancer cell line [12] to detect cancer-relevant proteins as potential biomarkers. In the current study, we analyzed plasma proteomes using an isotope-coded affinity tagging (ICAT) technique. This method has been developed to analyze relative amounts of cysteine-containing peptides in tryptic digests of protein extracts [6,13]. All the plasma samples used were depleted of six high-abundance plasma proteins by affinity chromatography. The biomarker candidates discovered were then confirmed and verified with pooled or individual samples, and further with a blinded set of multiple samples by Western blot assays. The employed ICAT and Western blot assay strategy enabled us to identify and quantify biotinidase (BTD) as a potential breast cancer biomarker in plasma.

## Methods

### Subjects

Blood samples were collected from breast cancer patients and normal healthy volunteers at the Seoul National University Hospital (Seoul, Korea). The use of human samples for research purpose was authorized by the Institutional Review Board of Seoul National University Hospital, and all the patients and volunteers agreed to take part in the experiment with the name signed on the informed consent document. The plasma sample was depleted of top six abundant serum proteins using a multiple-affinity MARS column (Agilent Technologies, Palo Alto, CA, USA) [12], and precipitated with trichloroacetic acid. The pellet was dissolved in ICAT denaturation buffer (6 M urea, 0.05% SDS, 5 mM EDTA, 50 mM Tris-HCl, pH 8.3).

### ICAT labeling and sample preparation

A pooled plasma sample from 6 breast cancer patients was labeled with a 'heavy (H)' ICAT reagent (Applied Biosystems, Framingham, MA, USA), whilst another pooled sample from 6 normal healthy women was labeled with a 'light (L)' reagent. We pooled equal amount of proteins from individual samples. Proteins (100 μg) in the denaturantion buffer were first reduced with 250 mM tris(2-carboxyethyl)phosphine for 30 min. ICAT-labeling was then performed using 350 nmol ICAT reagent with gentle shaking for 2 hr at 37°C, and terminated with 1.75 μmol DTT for additional 5 min. The H- and L-ICAT-labeled samples were mixed, diluted 10 fold with 50 mM Tris (pH 8.0), and digested with 5 μg of trypsin (Promega, Madison, WI, USA) for 16 hr at 37°C. The reaction was quenched at 0.5% phosphoric acid. The tryptic digest was applied on a polysulfoethyl A column (Western Analytical, Murrieta, CA, USA) equilibrated with 10 mM KH_2_PO_4 _in 25% ACN (pH 3.0) using an ÄKTA Explorer system (GE Healthcare Biosciences, Uppsala, Sweden), eluted with a 40-min gradient from zero to 0.4 M KCl, and collected on 40 fractions. The SCX fractions were neutralized by the addition of 10 volumes of 2× PBS, loaded on an ICAT^R ^avidin-catridge (Applied Biosystems), and then washed with PBS followed by 50 mM ammonium bicarbonate in 20% methanol, pH 8.3. ICAT-labeled peptides were eluted with a solution of 0.4% TFA in 30% acetonitrile, dried in vacuo, redissolved in 90 μl of 95% TFA, incubated at 37°C for 2 hr to cleave off the biotin moiety from the ICAT label, and finally dried again.

### Liquid chromatography and tandem mass spectrometry

An Agilent nanoflow-1200 series HPLC system was connected to a linear ion trap mass spectrometer (LTQ, Thermo Electron, San Jose, CA, USA). The dried ICAT-labeled peptide sample was reconstituted with 20 μL of 0.4% acetic acid, and an aliquot (1 μL) was injected to a reverse-phase Magic C18aq column (13 cm × 75 μm) equilibrated with 95% buffer A (0.1% formic acid in H_2_O) + 5% buffer B (0.1% formic acid in acetonitrile). The peptides were eluted in a linear gradient of 10 to 40% acetonitrile over 75 min. The MS survey was scanned from 300 to 2000 *m/z*, and followed by three data-dependent MS/MS scans with the following options: isolation width, 1.5 *m/z*; normalized collision energy, 25%; dynamic exclusion duration, 180 sec.

### Database searches

Peak lists were generated using Extract-msn program in Bioworks package v3.1 (Thermo Electron) with the following parameters: minimum ion count threshold, 15; minimum intensity, 100. The peak lists were compared against the human International Protein Index database including known contaminants (IPI, versions 3.24, European Bioinformatics Institute, http://www.ebi.ac.uk/IPI) using the SEQUEST (TurboSequest version 27, revision 12) allowing two missed cleavages (trypsin) and ±0.5 and ±3 Da mass tolerance for MS/MS and MS respectively. ICAT option (+227.26 Da fixed modification plus +9 Da variable modification) on cysteine residue was used and a variable modification of methionine oxidation (+16 Da) was allowed. Peptide assignment and quantification were performed with the Trans-Proteomic Pipeline provided by Institute for Systems Biology (TPP, version 4.0, http://www.proteomecenter.org). The SEQUEST search output was used as an input for Peptide-Prophet module and peptides with probabilities greater than 0.05 were included in the following Protein-Prophet. Proteins with probabilities greater than 0.5 were put into manual inspection to evaluate MS/MS spectral quality [14]. False discovery rate was 10% at the cut-off value of 0.5 before manual inspection. From a list of 238 proteins, 30 proteins were removed due to lack of quantification information and 53 proteins were removed due to their unreliable mass spectra during manual inspection. The number of removed proteins (53 ea) by manual inspection exceeded the number of estimated false positives (~24 ea). As a result, the false discovery rate for the final data set containing 155 proteins would be almost zero.

### Western blot analysis

Plasma samples were resolved on 10% SDS-PAGE gel, and electro-transferred to nitrocellulose membrane (Bio-Rad Laboratories, Hercules, CA, USA). In order to handle a large number of samples that exceeded loading sites of a gel, two or more gels were placed on a transfer membrane to minimize experimental bias of western blots. Immunobloting analyses were performed as described previously [12], using antibodies against neural cell adhesion molecule L1 (CHL1; Atlas, Stockholm, Sweden), α1-acid glycoprotein 2 (ORM2; Proteintech Group Inc., Chicago, IL, USA), monocyte differentiation antigen CD14 (CD14; Abcam, Cambridge, MA, USA), BTD (GeneTex Inc., San Antonio, TX, USA), and glutathione peroxidase 3 (GPX3; Abcam).

### Analysis of gene expression microarray data

Large cohort tissue microarray datasets of breast cancer patients analyzed by Human Genome U133A platform (GPL96) were downloaded from the Gene Expression Omnibus (GEO) database (http://www.ncbi.nlm.nih.gov/projects/geo/). The samples included 1,715 cases of biopsied breast cancer tissues (GSE1456, GSE2034, GSE2990, GSE3494, GSE4922, GSE5364 and GSE11121) and 95 cases of laser-capture microdissected (LCM) breast cancer tissues (GSE5847). The latter 95 samples were considered to be positive controls for breast cancer, since the LCM would be effective to get rid of contamination of normal tissues or blood cells in the breast cancer biopsies. As references, 39 microarray data of normal human breast tissues analyzed by the same or upgrade version (GPL570) were obtained from the GEO database. The obtained microarray data were analyzed by the R-package 2.7.2 using an Affy package and a gcRMA package to make normalization and adjustments of the background and average intensities. The average values of gene expression were calculated from replicate probes. From the log2-transformed values of each tumor sample, the log2 medians of included normal samples were subtracted to calculate a tumor-to-normal ratio (fold change) conveniently.

### Statistical analysis

Band intensities of Western blot images were quantified using ImageQuant version 5.2. (GE Healthcare Biosciences), and compared by Wilcoxon rank-sum test using SPSS 12.0 (SPSS, Chicago, IL, USA) due to the small sample size. For the statistical analysis of gene expression data, Wilcoxon rank-sum tests were performed with a 95% confidence interval, as the sample size of breast cancer tissues was greater than the size of normal controls and also the two data sets had been collected independently from large cohort breast cancer studies and standard human tissue microarray studies.

## Results

### Profiling of breast cancer plasma proteins by ICAT

**Figure 1:**
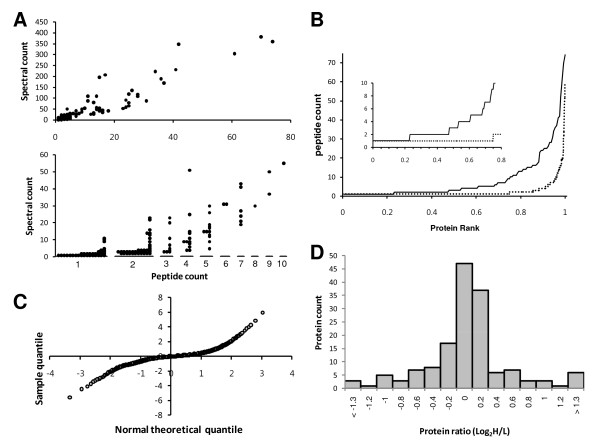
**Profiling of breast cancer plasma proteins by ICAT**. **(A) **Spectral count (total number of peptides) as a function of peptide count (number of unique peptides). Lower part is an enlarged view of spectral count ≤ 10 *vs*. peptide count. **(B) **Peptide count as a function of normalized rank of quantified proteins. Solid line: current data set, dashed line: tissue extract data set retrieved from [15] and reprocessed. **(C) **A quantile-quantile plot of the peptide quantification distribution. The percentiles of the logarithms of heavy-to-light peptide ratios are plotted against the percentiles from a normal distribution. **(D) **Profile of protein ratios. The ratios are log2-transformed. Bin size: 0.2.

The ICAT method was introduced for profiling of differentially expressed proteins in a set of pooled plasma samples of breast cancer patients (*n *= 6, age = 36 - 59, cancer grade = I - III) and age-matched normal healthy women (*n *= 6), as shown in Table 1. Analyzing the ICAT-labeled tryptic peptides by LC-MS/MS, a total of 155 proteins were confidently identified by matching MS/MS data (646 unique peptides) to the peptide sequences in human IPI database. Of these, 121 proteins (78%) were identified by two or more peptide matches, and 34 proteins (22%) were identified by single peptide match (Additional files 1 and 2). For the identified proteins, spectral count (total number of peptides) was plotted against peptide count (number of unique peptides; Figure 1A). About 60% of the proteins were identified based on 1-4 unique peptides and only 42% were identified based on 1-4 scans. The data were compared with another set of ICAT experimental data reported by Aebersold group in which tissue extract was analyzed [15]. Unlike the tissue extract data in which 75% of the proteins were identified with single peptide match, only 22% proteins were identified with single peptide match in our plasma data (Figure 1B). In contrast, the number of proteins identified by at least 5 peptides was similar: 62 for the tissue extract and 63 for the plasma sample. For each peptide count, spectral counts of plasma data distributed in wider range than those of the tissue extract data. Therefore, total number of identified proteins decreased in the current plasma data as the number of single peptide match decreased.

**Table 1:** Histopathological characteristics of breast cancers and normal healthy controls

Characteristics	Discovery by ICAT & qualification	Verification in a blinded set
Normal healthy control		
No. cases	6	21
Age (median)	36-51(49.5)	17-49 (35)
Breast cancer		
No. cases	6	21
Age (median)	36-51 (49.5)	36-78 (50)
Histological grade	Grade 1 (2), Grade 2 (2), Grade 3 (2)	Grade 0 (5), Grade 1 (4), Grade 2 (9), Grade 3 (2), Grade 4 (1)
Tumor size	T1 (3), T2 (3)	T0 (6), T1 (6), T2 (8), T3 (1)
Lymph node status	N0 (4), N2 (1), N3 (1)	N0 (12), N1 (7), N2 (2)
Metastasis	M0 (6)	M0 (20), M1(1)
Estrogen receptor	Negative (6)	Negative (10), Positive (10)*
Progesterone receptor	Negative (6)	Negative (12), Positive (8)*

* Receptor type for one breast cancer patient was not determined.

The ratios of differentially expressed proteins between breast cancer (heavy isotope-labeled) and normal healthy (light isotope labeled) plasma samples were calculated by the XPRESS software. Logarithm of all heavy-to-light (H/L) peptide ratios showed a maximum around zero (data not shown). A normal quantile plot (QQ plot) in which the percentiles of the logarithms of H/L peptide ratios are plotted against the percentiles from a normal distribution revealed that the distribution of peptide ratios was deviated from normality and had long tails at both ends (Figure 1C). Heavy-tailed distribution was also observed in protein ratios, indicating that the ICAT quantification data were appropriate for detecting proteins having significant abundance change between cancer and control. The apex of the logarithm of all H/L protein ratios was also around zero (Figure 1D). Thus, as shown in Additional file 3, most proteins showed the H/L ratios near one. In contrast, 33 proteins showed significantly different levels between plasmas of breast cancer patients and normal healthy control by more than 1.5 fold (Figure 1D). Of these proteins, 22 proteins showed increased abundance, and 11 proteins showed decreased abundance.

### Confirmation of protein abundance difference by Western blot analysis

We next chose five proteins (CHL1, ORM2, CD14, BTD and GPX3) among those that showed abundance change and exploited Western blot for the follow-up studies (Table 2). The selection was mainly based on gene ontology data about biological functions and disease relatedness of the proteins as well as the availability and specificity of antibodies commercially available. We first analyzed the pooled plasma samples that had been used for ICAT experiment. Of the five proteins tested, four except CHL1 displayed similar expression patterns with the ICAT ratios. The level of ORM2 determined by Western blot increased by 2.05 fold in the pooled plasma of breast cancer, whereas the ICAT ratio was 1.85 relative to that of normal healthy control. The BTD level, which decreased 2.3 fold in the ICAT experiment, showed 1.96 fold decrease in the Western blot analysis. The relative fold-changes of CD14 and GPX3 were also similar in ICAT and Western blot results. However, CHL1 showed reversed expression change, which hindered further verification.

**Table 2:** List of proteins and their abundance ratios between cancer and control

Uniprot*	Gene name	Abundance ratio (cancer/control)			
		
		ICAT (*N *= 2)^†^	Western blot (*N *= 2)^†^	Western blot (*n *= 12)^‡^	Western blot (*n *= 42)^‡^
O00533	CHL1	2.05	0.66		
P19652	ORM2	1.85	2.05	1.01	
P08571	CD14	1.69	1.29	1.78	
P43251	BTD	0.44	0.51	0.43	0.52
P22352	GPX3	0.37	0.53	0.42	1

*Uniprot, protein accession number in the SWISS-PROT/TrEMBL database.

^†^*N *denotes pooled plasma samples of breast cancer (*n *= 6) and normal healthy control (*n *= 6)

^‡^*n*, the number of individual samples.

**Figure 2:**
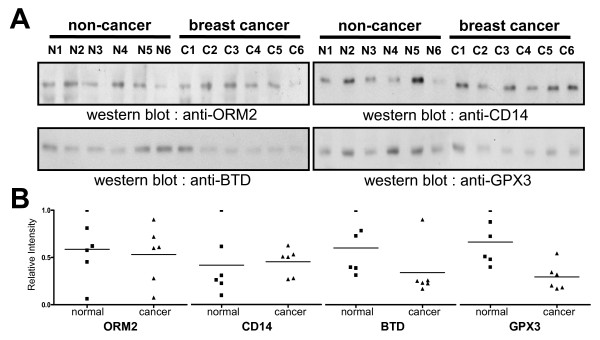
**Western blot analysis of ORM2, CD14, BTD and GPX3**. **(A) **Western blot analysis was performed for the plasmas of 6 breast cancer patients and age-matched 6 normal healthy women that had been previously used for ICAT experiment. **(B) **Western blot images were quantified by densitometric scanning and plotted. Lines denote the average of intensity values.

When the 12 samples were assayed individually for the four biomarker candidates by Western blots, expression levels of BTD and GPX3 were similar to those in the pooled samples (Figure 2A). But, the results of CD14 and ORM2 were significantly different from the data for pooled samples (Figure 2B).

### Gene expression of biomarker candidates in breast cancer tissues

**Figure 3:**
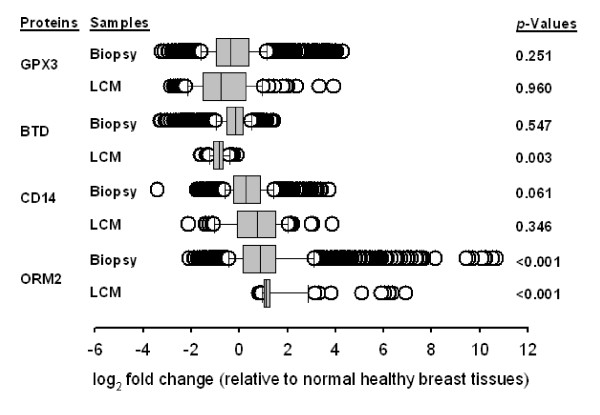
**The mRNA levels of ORM2, CD14, BTD and GPX3**. Box-plots of log2-transformed average fold changes (log_2_avFC) of the gene expression levels of BTD, CD14, GPX3 and ORM2 in breast cancer tissues (total *n *= 1,810) relative to the normal healthy tissues (*n *= 39). Statistically significant difference between breast cancer and normal tissues were tested by Wilcoxon tests. Breast cancer tissues were categorized into two groups of breast biopsy samples (Biopsy, *n *= 1,715) and laser capture microdissected samples (LCM, *n *= 95).

To investigate changes in mRNA levels of BTD, CD14, GPX3 and ORM2 in breast cancer tissues, a total of 1,849 microarray raw data (CEL files) was created by using Affymetrix U133A (GPL96) and U133+2.0 (GPL570) platforms were downloaded from the GEO database. Among the four biomarker candidates, ORM2 showed significant up-regulation in both biopsied and LCM breast cancer tissues relative to normal controls (*p *< 0.001), whose log_2_-transformed fold changes (log_2_FC) in the medians were 0.88 and 1.09, respectively (Figure 3). BTD displayed a significant change only in the LCM samples (log_2 _FC = -0.86, *p *= 0.003), but not in the biopsied samples (log_2_FC = -0.18, *p *= 0.547). However, the medians of CD14 and GPX3 in breast cancer patients did not have significant difference with the normal controls (*p *> 0.05). The LCM microarray data of BTD showed a greater mRNA fold change than the biopsied tissue microarray data. This implies that the down-regulated gene expression of BTD was strongly associated with cancer cells.

### Verification of BTD as potential breast cancer biomarker in plasma

**Figure 4:**
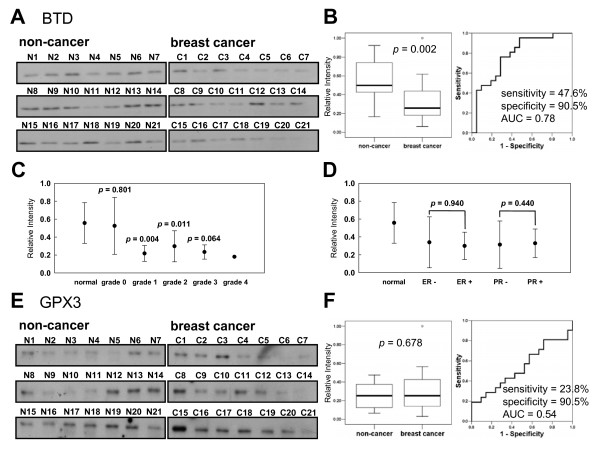
**Western blot analysis of BTD and GPX3 in a blinded set of plasmas**. **(A, E) **Western blot images of BTD and GPX3 in a blinded set of plasmas from 21 breast cancer and 21 normal healthy women. **(B, F) **Box-plots (left panels) and receiver operating characteristic (ROC) curves (right panels). **(C) **BTD levels according to breast cancer grade. **(D) **BTD levels according to estrogen receptor (ER) and progesterone receptor (PR) status.

In the initial stages of biomarker discovery using ICAT and Western blot analysis, we confidently observed that BTD and GPX3 were significantly down-regulated in breast cancer plasma compared to age-matched normal healthy control. For the clinical use, they must be verified in a larger sample size. As shown in Table 1, a blinded set of plasmas from 21 breast cancer patients (age = 36 - 78, cancer grade = O - IV) and 21 normal healthy women (age = 17 - 49) were tested to determine individual levels of BTD and GPX3 by Western blots. Consistent with the preliminary data, significant down-regulation of BTD was observed in breast cancer plasma compared to the normal healthy control (*p *= 0.002; Figure 4A). The median value of BTD in breast cancer was 1.9 fold lower than that of normal healthy women (Figure 4B). BTD levels were significantly lower in breast cancer grade I - IV than normal healthy controls, but the BTD level of cancer grade O was not (*p *= 0.801; Figure 4C). Estrogen receptor status (*p *= 0.940) and progesterone receptor status (*p *= 0.440) were not associated with the plasma BTD levels (Figure 4D). Dividing the cancer patients equally into two subgroups by the age, the difference between the BTD levels of younger and older groups was not statistically significant (*p *= 0.888). Neither significant difference was observed in case of the healthy control (*p *= 0.481). The analysis of a receiver operating characteristic (ROC) curve showed that the area under the ROC curve (AUC) reached 0.78 (sensitivity = 47.6%; and specificity = 90.5%), suggesting it as a potential breast cancer biomarker in plasma. In case of GPX3, however, there was no significant difference between the medians of breast cancer and normal healthy women (*p *= 0.678; Figure 4E), indicating that GPX3 cannot critically discriminate breast cancer from normal healthy control. Taking these results into account together, BTD is considered to be a novel potential biomarker for breast cancer.

## Discussion

In this study we discovered serum BTD as a potential breast cancer biomarker through the biomarker development pipeline encompassing mass spectrometry based screening and independent downstream immunoblot assays. Biomarker candidates discovered by ICAT analysis of plasmas from 6 breast cancer patients and 6 age-matched normal healthy controls were examined by Western blot in the same sample set. The two candidates, BTD and GPX3, confirmed by this approach were next tested with immunoblot assay in a blinded set of breast cancer and control to ascertain the markers ability to differentiate the two groups.

The ICAT method applied here for the screening of differentially expressed proteins has low-throughput and is not suitable for a large number of samples. Therefore, a sample pooling strategy was employed to overcome this drawback. Although pooling reduces the expense of costly assays, nevertheless their still remains a possibility to obtain biased quantification result stemming from individual variations, which necessitates an independent downstream assay in individual samples. In the immunoblot assays after the ICAT discovery phase, we had to drop some candidate markers like GPX3. Our results suggest that the sample pooling strategy has both advantages and disadvantages. We adapted immunodepletion of top six high-abundance proteins to dig deep into low abundance proteins since plasma proteins are present over a wide dynamic range in concentration. This antibody-based separation system has demonstrated high efficiency to remove the specifically targeted proteins as well as both reproducibility and selectivity [16-19]. Actually, it was effective in our study enough to detect protein that exist at about a few μg/ml in plasma such as L-selectin [20].

Differential profiling by ICAT method enabled us to identify and quantify a total of 155 plasma proteins. The number was much smaller than that of proteins identified by the same method with tissue extract proteome [15], which was accounted for by the small number of the proteins identified based on single peptide match. On the contrary, spectral counts for each peptide count were distributed more widely: for example, spectral counts ranged from 2 to 23 for the proteins identified with two unique peptides. This is clearly due to the concentration depth of plasma proteome. Our result indeed covered 4 orders of magnitude from the lowest L-selectin at 0.67 μg/ml to the highest α-2-macroglobulin at 1.8 mg/ml [20]. The lower detection limit was similar to another study performed on plasma proteome [21], which indicated that ICAT analysis for immunodepleted plasma samples is effective for biomarker discovery medium-abundance protein. Comparing current plasma proteome list with those of our previous studies [11,12] and a recent report related to multiplex serum biomarker [22], several proteins, such as transthyretin, vitamin D-binding protein, and endorepellin were commonly identified. However, in the current study, these proteins showed little change among breast cancer patients and normal healthy controls (Additional file 1), and hence were excluded from further analysis. This is due to the difference in sample sources and in the screening method. 2DE or SELDI-TOF was used in the previous studies while we adapted ICAT strategy in this study. As mentioned earlier, protein quantification by ICAT is based only on cysteine containing peptide whereas 2DE reflects whole protein features including proteolytic processing. In addition, the previous study also could not draw decisive conclusion about discriminatory power of transthyretin and vitamin D-binding protein [22]. In case of endorepellin, we discovered LG3 fragment of the protein as a biomarker in the previous study [12]. In contrast, the single ICAT peptide matched to endorepellin covered other part than the LG3 fragment in the current study. Moreover, common proteins such as heptoglobin, serum albumin and transferrin were detected not due to their differential regulation in breast cancer but due to incomplete depletion of these proteins from plasma during immunoaffinity chromatography (in this study) or incomplete removal of blood vessels in the preparation of breast tissues (in the previous study [11]). Therefore, such proteins were not put into the next qualification phase of this study, while the proteins chosen for qualification were seemingly novel proteins discovered in relation to breast cancer.

Among the proteins showing differential level between breast cancer patients and normal healthy women, we chose five proteins for further verification. In this experimental flow, 4 biomarker candidates including ORM2, CD14, BTD and GPX3 showed relatively similar quantification results both in ICAT and Western blot. In case of CHL1, we observed an inconsistency between two quantification results (Table 2). According to ICAT result, CHL1 was quantified based on a single peptide (LHMLELHCESK; Additional file 2) in which the methionine residue had formerly been oxidized. Methionine oxidation occurs frequently during sample preparation and handling. Therefore, it seems inappropriate to quantify proteins based on a single oxidized peptide. Increased level of ORM2 [23] and CD14 [24] or down regulation of GPX3 [25] appears to be reasonable when interpreted in relation to their biological functions. However, we could not confirm their ICAT fold changes in the subsequent verification steps. It is likely that expression levels of ORM2, CD14 and GPX3 vary with age or with other factors that are currently unknown. Other possibility like stochastic variation cannot be excluded. Interestingly, the mRNA level of BTD in breast cancer tissue also changed compared to normal breast tissues. There is no evidence and it is unlikely that expression change of BTD in breast cancer tissues affect their levels in plasma. Therefore, the abundance change of BTD observed in the plasmas of breast cancer patients need not necessarily be the same as the abundance change of tissue mRNA. Nevertheless, it is worthy investing BTD for their potential use as tissue biomarkers in future study.

Among five candidates, BTD subsisted until the last step of verification of the biomarker pipeline. BTD is known to catalyze the release and recycling of endogenous biotin [26]. It is known to be secreted into blood circulation [27], and is highly active in the serum, liver, kidney and adrenal glands. Its deficiency results in various diseases such as seizures, hypotonia, hyperammonemia, and so forth [28]. We first reported here that its level was consistently down-regulated in breast cancer plasma. It is, yet, difficult to explain how BTD is down-regulated in breast cancer plasma. Interestingly, transcriptional levels of BTD in breast cancer tissues were also down-regulated. So, it merits further studies to elucidate down-regulation of BTD in relation to the behavior of breast cancer cells. Despite many uncertainties in the cellular and molecular mechanism of BTD, it is apparent from our results that BTD is down-regulated in breast cancer plasma. It will be useful and applicable for clinical use alone or in combination with other biomarkers in detecting breast cancer by less invasive techniques using plasma samples.

## Conclusion

Blood is a containment of communicating cells, tissues and organs in human body. Physiological change due to occurrence of a certain disease will be reflected to the blood proteome. An increased or decreased protein in the plasma can be considered to be a serological biomarker for the disease. In the present work we adapted comparative proteomic study using ICAT labeling and tandem mass spectrometry in search of new serological biomarkers for breast cancer. Among 33 proteins that showed differential abundance level between plasmas of breast cancer patients and normal healthy controls, BTD was shown to be decreased in breast cancer plasma. BTD successfully discriminated breast cancer patients from normal healthy controls in a blinded set of 42 cases. Our study suggests a diagnostic value of serum BTD for the detection of breast cancer.

## Competing interests

The authors declare that they have no competing interests.

## Authors' contributions

UBK performed all studies and drafted the manuscript. YA performed Western blot analysis. JWL collected the plasma samples used in this study. YHK downloaded and analyzed GEO microarray data. JK and MHY provided analytical platforms. DYN and CL designed the study. CL revised the manuscript. All authors were involved in the conception of the study and data interpretation. All authors have read and approved the final manuscript.

## Pre-publication history

The pre-publication history for this paper can be accessed here:

http://www.biomedcentral.com/1471-2407/10/114/prepub

## Supplementary Material

Additional file 1 **Plasma proteins identified and quantified by ICAT method**. Table of all plasma proteins identified and quantified by ICAT method including IPI database accession number, protein coverage, number of unique peptides and ICAT fold-ratio. Click here for file

Additional file 2 **Peptides identified by ICAT and LC-MS/MS**. List of peptides identified by LC-MS/MS and SEQUEST database search. Click here for file

Additional file 3 **MS spectra and chromatograms of three typical proteins which showed no significant changes in breast cancer**. MS/MS spectra of unique peptides **(left panels) **and their ICAT-labeled parent ion chromatograms **(right panels)**. Heavy and light ICAT-labeled peptides are shown with gray- and black-colored peaks, respectively. **(A) **Alpha-2-HS-glycoprotein with unique peptide sequence, C^§^NLLAEK. **(B) **A vitamin D-binding protein with VC^+^SQYAAYGEK. **(C) **A histidine-rich glycoprotein with VSPTDC^+^SAVEPEAEK. C^§ ^and C^+ ^are cysteines labeled with a heavy ^13^C-reagent and a light ^12^C-reagent, respectively. Click here for file
