# Supplementary figures and images for: Differential profiling of breast cancer plasma proteome by isotope-coded affinity tagging method reveals biotinidase as a breast cancer biomarker
Source: BMC Cancer. 2010 Mar 26;10:114. doi: 10.1186/1471-2407-10-114 (PMC2861033; doi:10.1186/1471-2407-10-114)

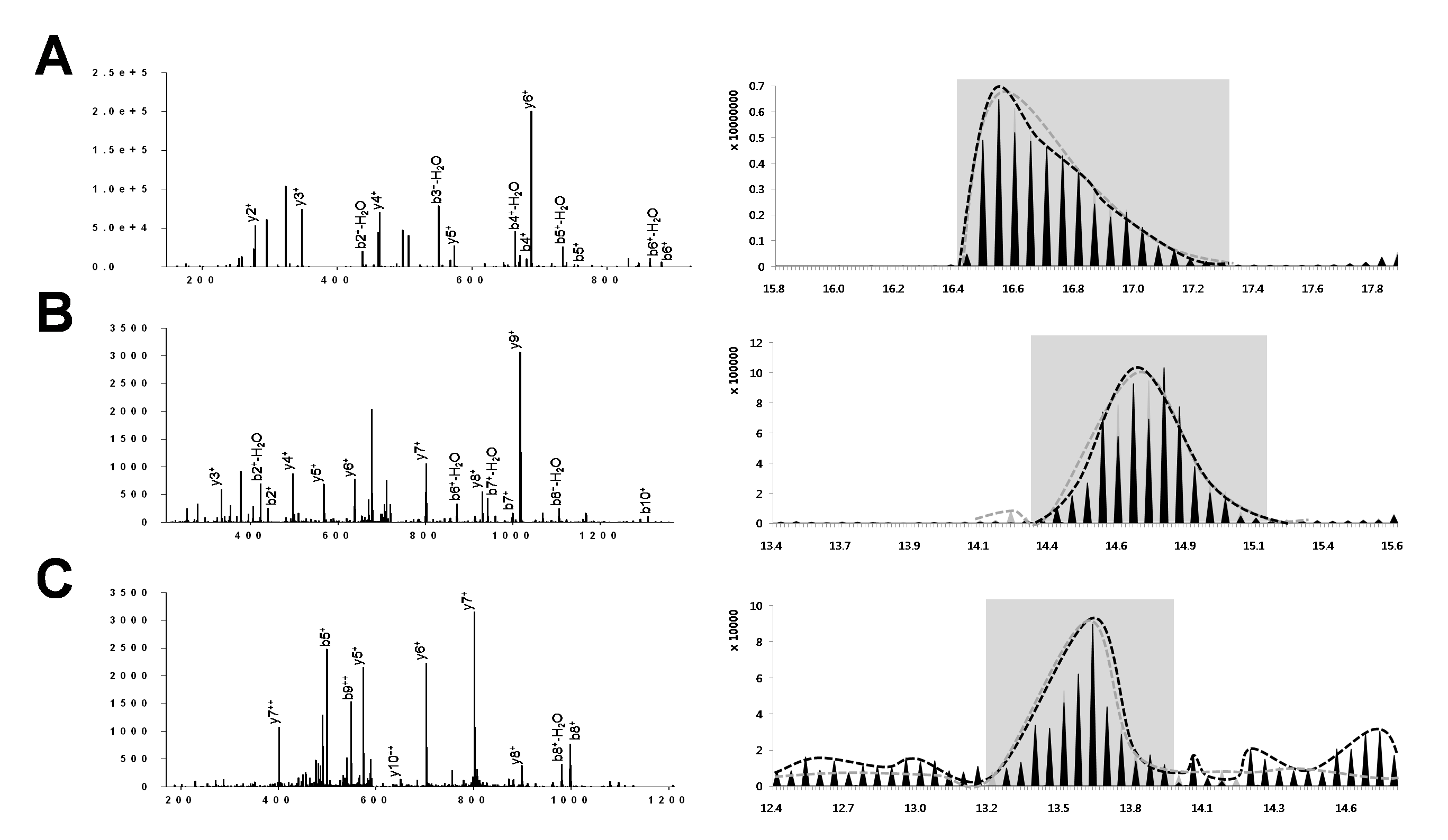

Supplement: Additional file 3 — MS spectra and chromatograms of three typical proteins which showed no significant changes in breast cancer. MS/MS spectra of unique peptides (left panels) and their ICAT-labeled parent ion chromatograms (right panels). Heavy and light ICAT-labeled peptides are shown with gray- and black-colored peaks, respectively. (A) Alpha-2-HS-glycoprotein with unique peptide sequence, C§NLLAEK. (B) A vitamin D-binding protein with VC+SQYAAYGEK. (C) A histidine-rich glycoprotein with VSPTDC+SAVEPEAEK. C§ and C+ are cysteines labeled with a heavy 13C-reagent and a light 12C-reagent, respectively. [file 1471-2407-10-114-S3.PNG]
